# SOCS3 blocks HIF-1α expression to inhibit proliferation and angiogenesis of human small cell lung cancer by downregulating activation of Akt, but not STAT3

**DOI:** 10.3892/mmr.2015.3368

**Published:** 2015-02-17

**Authors:** JUN WAN, YUN CHE, NINGNING KANG, WEI WU

**Affiliations:** 1 Departments of Thoracic Surgery, The First Affiliated Hospital of Anhui Medical University, Hefei, Anhui 230022, P.R. China; 2 Departments of Hematology, The First Affiliated Hospital of Anhui Medical University, Hefei, Anhui 230022, P.R. China

**Keywords:** suppressor of cytokine signaling 3, hypoxia-inducible factor-1α, vascular endothelial growth factor-A, signal transducer and activator of transcription 3, Akt, angiogenesis potential, proliferation, small cell lung cancer NCI-H446 cells

## Abstract

Suppressor of cytokine signaling 3 (SOCS3) is a major negative regulator of signal transducer and activator of transcription 3 (STAT3) during tumorigenesis. Previous studies have indicated that SOCS3 also regulates other signaling pathways, such as PI3K/Akt. However, little is known about the specific molecular mechanisms by which SOCS3 regulates the proliferation and angiogenesis of small cell lung cancer (SCLC) cells. The present study investigated the effect of SOCS3 upregulation on the expression of hypoxia-inducible factor-1α (HIF-1α) and how this affects the proliferation and angiogenesis of SCLC cells. It was investigated whether this interaction is associated with STAT3 or the Akt signaling pathway. The results of the present study revealed that SOCS3 negatively regulates proliferation and angiogenesis of NCI-H446 cells and that HIF-1α is required in this process. The results also suggested a suppressive role of SOCS3 in Akt signaling, but not STAT3 signaling to block HIF-1α expression and a previously unidentified regulatory mechanism for Akt function. In conclusion, the present study suggested that SOCS3 targets the Akt signaling pathway to inhibit HIF-1α expression and affect the growth and angio-genesis of SCLC cells, and may therefore be considered as a potential novel therapeutic for the treatment of SCLC.

## Introduction

Hypoxia inducible factor-1α (HIF-1α) may regulate the expression of numerous cytokines, such as vascular endothelial growth factor-A (VEGF-A), and promote the proliferation (1) and the angiogenic potential of small cell lung cancer (SCLC) (2). The results of previous studies indicated that HIF-1α may be an effective molecular therapeutic target for SCLC (3,4). Several different approaches were used to effectively inhibit HIF-1α expression in tumors, including anti-sense oligonucleotides against HIF-1α (5), a dominant-negative form of HIF-1α, and a peptide, which inhibits the binding between HIF-1α and the coactivator p300/CBP (6). Inhibiting HIF-1α expression by increasing the expression of certain anti-tumor factors acts as a therapeutic tool for the molecular treatment of tumors (7).

Suppressor of cytokine signaling 3 (SOCS3) is a member of the SOCS family (8) and is structurally composed of distinct functional domains, which include an N-terminal kinase inhibitory region, a central SH2 domain and a C-terminal homologous region termed the SOCS-box (9). SOCS3 has been reported to be silenced in certain tumors (10). SOCS3 expression is suppressed due to aberrant methylation in its promoter region, which frequently occurs in a variety of human tumors (11,12). Restoring the genetic function of the SOCS3 gene by demethylation or SOCS3 gene transfection was shown to suppress tumor cell growth by attenuating activation of signal transducer and activator of transcription 3 (STAT3) in several human cancer cell lines (13). However, apart from STAT3, little is known about the mechanism by which SOCS3 modulates other intracellular signaling cascades, such as PI3K/Akt, which also has a critical role in tumorigenesis and in turn affects the biological characteristics of malignant tumors, including angiogenesis, migration and proliferation (14). The PI3K/AKT pathway has a critical role in multiple cellular functions, including metabolism, proliferation, growth and survival (15). There is growing evidence that this pathway is frequently deregulated during tumorigenesis, which in turn affects certain biological phenotypes (16).

The present study explored the effect of SOCS3 restoration by gene transfection or demethylation on HIF-1α expression and proliferation of SCLC cells, and assessed the involvement of Akt and STAT3 signaling in these processes. The results indicated that SOCS3 may be considered as a novel therapeutic for treating SCLC.

## Materials and methods

### Ethics

The present study was approved by the Institutional Review Board of Biomedicine of Anhui Medical School (Hefei, China).

### Cell culture and experimental treatment

The human SCLC cell line NCI-H446 (Cell Bank of Shanghai Institutes for Biological Sciences, China Academy of Sciences, Shanghai, China) was cultured as in a previous study (1). Once passaged to the fifth generation, the cells were grown in 75 cm^2^ culture flasks and harvested in a solution of trypsin-EDTA at the logarithmic growth phase. A cytometry assay was used for cell counting (2). For the demethylation treatment, NCI-H446 cells (60–70% confluence) were treated with 5 *μ*m 5-aza-2′-deoxy-cytidine for six days and the medium was replaced every other day. DNA, RNA and protein were extracted for analysis using kits obtained from Sangon Biotech Co., Ltd. (Shanghai, China). For blocking Akt expression, wortmannin (Sigma-Aldrich, St. Louis, MO, USA) was used as an inhibitor of the PI3K/Akt signaling pathway. Wortmannin was dissolved in dimethyl sulfoxide (DMSO; Sigma-Aldrich) and final concentrations of 20 *μ*M were used to treat the cells. Equal volumes of DMSO were used for the vehicle control (17). CPA-7, which is a specific inhibitor of the PI3K/Akt signaling pathway, was used for blocking STAT3 expression and dissolved in DMSO to achieve a final concentration of 10 *μ*M (18). For plasmid transfection, pcDNA3-STAT3 and pcDNA3-Akt were received from the Department of Viral-Gene Therapy, Shanghai Eastern Hepatobiliary Surgery Hospital (Shanghai, China).

### Adenovirus vector construction and cell transfection

Tumor-specific replication-defective adenovirus type 5 (Ad5) was used as the vector. Ad5-SOCS3-green fluorescence protein (GFP) construct was received from the Department of Viral-Gene Therapy, Shanghai Eastern Hepatobiliary Surgery Hospital (Shanghai, China). SOCS3 cDNA was amplified using gene-specific primers: Sense, 5′-GCGGTCGACATG TACCCATACGACGTCCCGATTACGCTATGGTCACCCA-CAGCAAG-3′ and antisense, 5′-GATGCGGCCGCTTA AAGCGGGGC-3′ (Sangon Biotech Co., Ltd.). This was designed to generate an N-terminal hemagglutinin (HA) tag and restriction sites *Sal*I and *Not*I at the 5′- and 3′-cDNA ends. A recombinant adenovirus (pAd5-SOCS3-HIF-1α) encoding human HIF-1a gene and human SOCS3 gene was contructed and then NCI-H446 cells were transfected. The HIF-1α gene which was amplified by polymerase chain reaction and the SOCS3 gene from the plasmid pIRES2-SOCS3 were both subcloned into the shuttle vector pShuttle-CMV. A shuttle vector pShuttle-SOCS3-HIF-1α was obtained and then it was co-transformed into adenoviral backbone plasmid pAd5 to make homologous recombination with the HIF-1α and SOCS3 genes. For transfection, cells were cultured in six-well plates and exposed to viral supernatant in the absence of cytokines and serum with different multiplicities of infection (MOI). A constructed recombinant adenovirus vector was used for NCI-H446 cell transfection.

### Methylation-specific polymerase chain reaction (PCR)

Methylation-specific PCR (MSP) was performed based on results of a previous study (9). The methylation-specific primer was designed using the Methyl Primer Express software v1.0 (Applied Biosystems, Foster City, CA, USA). Primer sequences are shown in Table I. The PCR reaction conditions were as follows: Initial denaturation at 95°C for 10 min; 40 cycles of denaturation at 95°C for 40 sec; annealing at 65°C for 30 sec and elongation at 75°C for 40 sec. Finally, cycling was completed with an elongation step at 75°C for 10 min. Sequences of MSP primers in exon 1 of SOCS3 were: Sense, 5′-TTCGAGGTGTTCGATTAGAC-3′ and antisense, 5′-AAAATGCTTCCGACATAGAT-3′. Sequences of MSP primers in intron 1 of SOCS3 were: Sense, 5′-GCCTCCGGGTAAGCGTGGATTAG-3′ and antisense, 5′-AATACATAGAGGCTGCGCGAAGC-3′.

### Reverse transcription-quantitative PCR (RT-qPCR) analysis

RT-qPCR was performed using a SYBR ExScript RTPCR kit (Takara Biotechnology Co. Ltd., Dalian, China) according to the manufacturer’s instructions and using the iCycler Real-Time PCR detection system (Bio-Rad, Hercules, CA, USA). All RNA samples were run in duplicate on 96-well optical PCR plates. The PCR conditions were set as follows: Initial incubation for 2 min at 50°C, denature at 95°C for 10 min, 32 cycles of 94°C for 40 sec, 60°C for 40 sec and 60°C for 40 sec, followed by 72°C for 1 min. PCR reaction conditions and primer sequences are shown in Table II. Relative changes in gene expression were calculated using the equation: Relative changes in gene expression =2^−ΔΔCT^ where ΔCt = Ct target - Ct β-actin and ΔΔCt = ΔCt Unmethylated - ΔCt control.

### Western blot analysis

Cells were harvested and analyzed for the protein levels of SOCS3, HIF-1α, STAT3, phosphorylated (p)STAT3, Akt and pAkt. Briefly, all proteins were extracted by disrupting cells in radioimmunoprecipitation lysis buffer (Beyotime Institute of Biotechnology, Haimen, China), separated on a 10% SDS-polyacrylamide gel (Beyotime Institute of Biotechnology) and then transferred onto a polyvinylidene difluoride (PVDF) membrane. The membranes were then blocked at room temperature for 1 h with 5% non-fat milk in Tris-buffered saline containing Tween 20 (TBST). Subsequently, the membranes were incubated with rabbit monoclonal anti-SOCS3 (1:400 dilution); rabbit monoclonal anti-HIF-1α (1:500 dilution); rabbit monoclonal anti-STAT3 (1:1,000 dilution), rabbit monoclonal anti-β-actin (1:1,000 dilution) and rabbbit monoclonal anti-Akt (1:1,000 dilution; Wuhan Boster Biological Engineering Technology Limited Company, Wuhan, China), rabbit monoclonal anti-phospho-Akt (1:1,000 dilution; Cell Signaling Technology, Beverly, MA, USA) or rabbit monoclonal anti-phospho-STAT3 (1:800 dilution; Santa Cruz Biotechnology, Austin, TX, USA) at 37°C for 2 h. The membranes were subsequently incubated with goat anti-rabbit horseradish peroxidase (HRP)-conjugated immunoglobulin G (Wuhan Boster Biological Engineering Technology Limited Company) at room temperature for 1 h. Immunoreactivity was detected using an enhanced chemiluminescence kit (EZ-ECL kit for HRP; Beyotime Institute of Biotechnology) and images of gels were captured on X-ray film (Shanghai Baiyun Sanhe Sensitive Materials Co., Ltd., Shanghai, China). β-actin was used as an internal control.

### Immunofluorescence

Once the cells were passaged to the fifth generation, a cell suspension (1×10^5^ cells/ml) was prepared, transferred to cover slips and incubated at 37°C in humidified atmosphere containing 5% CO_2_ and 20% O_2_ for 24 h. The cells were then fixed with 4% paraformaldehyde (Beyotime Institute of Biotechnology) for 20 min, permeabilized with 0.5% Triton X-100 (Beyotime Institute of Biotechnology) and incubated with anti-CD34 primary antibody (Wuhan Boster Biological Engineering Technology Limited Company) overnight at 4°C. Subsequently, cells were washed and incubated with Rhodamine Red-X secondary antibodies (1:1,000 dilution) (Wuhan Boster Biological Engineering Technology Limited Company) for 1 h at 37°C. Images were observed using a laser confocal microscope (LSM710; Carl Zeiss, Jena, Germany).

### In vivo tumor experiments and angiogenic measurements

A total of 36 male congenital athymic BALB/c nude mice were obtained from the Experimental Animal Center of the Shanghai Jiao Tong University School of Medicine (Shanghai, China). They were maintained under pathogen-free conditions in accordance with established institutional guidance and approved protocols. The mice were divided into three groups (n=12/group): Ad5 group, Ad5-SOCS3 group and Ad5-SOCS3+HIF-1α group. All experiments were performed using 6–8 week-old mice weighing 16–22 g. The mice were maintained under controlled light (12 h light/12 h dark) and temperature (22°C) conditions, were housed in individual cages and were given *ad libitum* access to standard mouse chow. Animal care and experimental procedures were performed with the approval of the Animal Care and Experimental Center of Anhui Medical University, under estanlished guidelines. The mice were sacrificed by cervical dislocation. *In vitro* cultured NCI-H446 cells (1×10^7^) transfected with Ad5-SOCS3 or empty Ad5 vector suspended in 100 ml PBS were subcutaneously injected into the flank area of mice. NCI-H446 cells (1×10^7^) without transfection were injected when tumors reached 3–5 mm in diameter. Mice were injected with either vehicle (10% DMSO/PBS), 4 mg/kg wortmannin or 5 mg/kg CPA-7 twice weekly. The tumor size was measured with calipers every three days and the tumor volume was calculated according to the formula: Volume = width^2^ × length × 0.5. Subsequently, tumors were removed and weighed 30 days following inoculation.

According to the method of QingXu (19), angiogenesis quantification was performed in a double-blinded manner and imaging software was used to capture bright-field images from two fields for each sample. Data for these image files were collected using Image-Pro Plus software (v. 5.0; National Institutes of Health, Bethesda, MD, USA). CD34-positive areas (blood vessels) were selected using the histogram and dropper tools. The Count function was then used to total the number of CD34-positive areas. Measurement was selected within the Count/Size function and the Area tool was utilized to calculate the area of CD34-positive blood vessels in each entire field. Every field in each of the entire tumor sections was examined and analyzed.

### Statistical analysis

SPSS 13.0 software (SPSS, Inc., Chicago, IL, USA) was used for data processing. An independent-samples t-test was used to evaluate the differences in optical density (OD) values or counting of tumor cells between groups with various treatments. All values are expressed as the mean ± standard deviation of three independent experiments. P<0.05 was considered to indicate a statistically significant difference.

## Results

### Demethylation does not markedly affect protein expression of SOCS3

**Figure 1:**
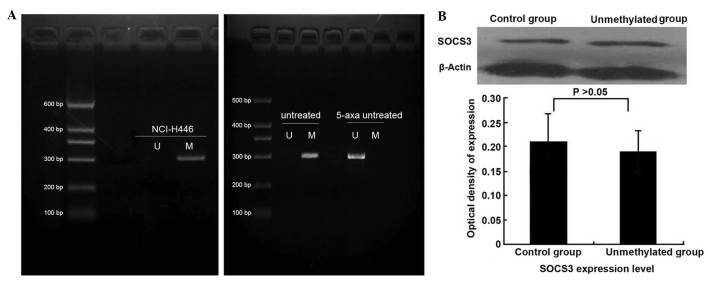
When treated with the demethylating agent 5-aza-2′-deoxycytidine, protein expression level changed little, although mRNA levels were significantly upregulated. (A) Methylation status of SOCS3 in NCI-H446 cells was detected. Visualized bands in lane M and lane U are methylated 310-bp products with methylation-specific primers and unmethylated 310-bp products with unmethylation-specific primers, respectively. Methylation was present in NCI-H446 cells. A visible band in lane U appeared in NCI-H446 cells following treatment with 5-aza-2′-deoxycytidine. This demonstrated that mRNA levels of SOCS3 were upregulated following treatment with demethylating agent. (B) Western blot detection of SOCS3 expression revealed minor changes in protein levels following treatment with 5-aza-2′-deoxycytidine, which were not significant. Values are normalized to β-actin. SOCS3, suppressor of cytokine signaling, 3.

SOCS3 mRNA expression was weak in NCI-H446 cells, but was markedly increased demethylation by treatment with 5-aza-2′-deoxycytidine (Fig. 1A). At the protein level, it was found that following treatment with 5-aza-2′-deoxycytidine, the expression of SOCS3 was not upregulated (Fig. 1B). This finding differed from results of previous studies, in which demethylation-associated upregulation of SOCS3 in A549 cells and squamous cell carcinoma cells of the head and neck was observed following treatment with 5-aza-2′-deoxycytidine (20).

### Transfection efficiency of SOCS3

**Figure 2:**
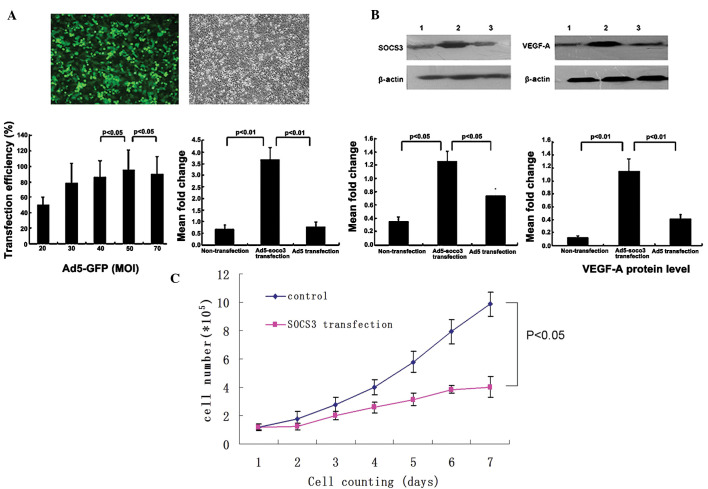
Transfection with Ad-SOCS3 upregulates the expression levels of SOCS3 in NCI-H446 cells and inhibits their proliferative and angiogenic potential. (A) Determination of transduction conditions and evaluation of SOCS3 induction efficiency. Five different MOIs (20, 30, 40, 50 and 70) were assessed in the transduction experiment (60 h). The transduction efficiency was the highest when the MOI was 50 (MOI 50 vs. MOI 40; P<0.05; MOI 50 vs. MOI 70; P<0.05). Transduction efficiency of NCI-H446 cells with Ad5-GFP after 60 h is shown in the fluorescent image (MOI 50; magnification, ×200). After the cells were transduced with Ad5 and Ad5-SOCS3 (MOI, 50), the mRNA expression levels of SOCS3 were measured using quantitative polymerase chain reaction (NCI-H446/Ad5-SOCS3 group vs. control group, P<0.01; NCI-H446/Ad5 group vs. NCI-H446/Ad5-SOCS3 group, P<0.01) (B) Western blot analysis of SOCS3 and VEGF-A protein expression. Representative images of three independent experiments (lane 1, SOCS3 and VEGF-A protein expression in the control group; lane 2, SOCS3 and VEGF-A protein expression in Ad5-SOCS3 transfection group; lane 3, SOCS3 and VEGF-A protein expression in Ad5 transfection group) and relative expression of SOCS3 and VEGF-A protein normalized to β-actin (SOCS3 expression in Ad5-SOCS3 transfection group vs. control group, P<0.05; SOCS3 expression in Ad5-SOCS3 transfection group vs. Ad5 transfection group, P<0.05; VEGF-A expression in Ad5-SOCS3 transfection group vs. control group, P<0.01; VEGF-A expression in Ad5-SOCS3 transfection group vs. Ad5 transfection group, P<0.01). (C) Growth curve of the cells in two groups. In the control group, NCI-H446 cells entered the period of logarithmic growth after the fourth day. Following transfection with Ad5-SOCS3, cell growth was significantly decreased and the logarithmic growth phase was not entered (NCI-H446/SOCS3 group vs. NCI-H446, P<0.05). Ad5, tumor-specific replication-defective adenovirus type 5; SOCS3, suppressor of cytokine signaling 3; VEGF, vascular endothelial growth factor; MOI, multiplicity of infection; GFP, green-fluorescent protein.

In the present study, cells were divided into 2 groups: Ad5-SOCS3-GFP transfection group and Ad5-GFP transfection group. The efficiency of transfection was estimated by determining the percentage of fluorescent cells in cells infected with Ad-SOCS3-GFP. The appropriate MOI was selected using the following formula: MOI = titer (pfu) × viral fluid (L)/cell number. Using fluorescence microscopy, it was observed that the transfection efficiency of the adenoviral vectors into cells was high and reached >90% at a MOI of 50. mRNA and protein levels of SOCS3 were significantly upregu-lated following transfection with Ad-SOCS3-GFP (Fig. 2A and B).

### Upregulation of SOCS3 inhibits the proliferative and angiogenic potential of SCLC

Following transfection with Ad-SOCS3, mRNA and protein expression of SOCS3 were upregulated in NCI-H446 cells (Fig. 2A and B). In addition the cell growth curve demonstrated that following transfection with Ad5-SOCS3-GFP, the cell proliferation rate was significantly inhibited, particularly from the fifth day (Fig. 2C). The expression of the angiogenic factor VEGF-A was found to be significantly inhibited following upregulation of SOCS3 (Fig. 2B).

### HIF-1α is required for SOCS3-mediated inhibition of proliferation and angiogenesis of SCLC cells

**Figure 3:**
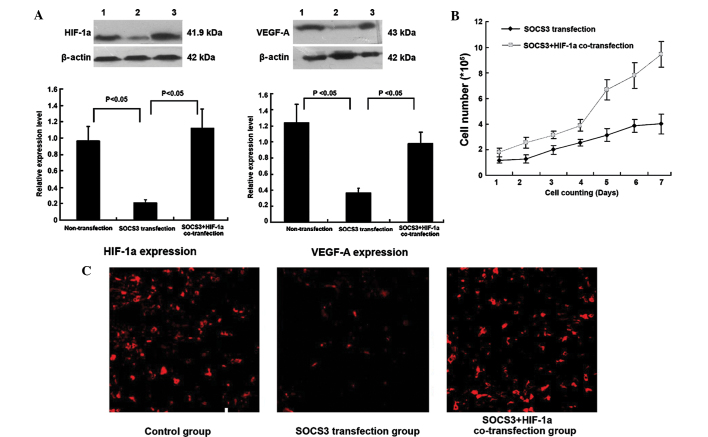
HIF-1α is required for proliferation of SOCS3-transduced SCLC cells and their angiogenic potential. (A) Western blot analysis of HIF-1α and VEGF-A protein expression. Representative images of three independent experiments (lane 1, HIF-1α and VEGF-A protein expression in the non-transfection group; lane 2, VEGF-A and HIF-1α protein expression in SOCS3 transfection group; lane 3, HIF-1α and VEGF-A protein expression in SOCS3 and HIF-1α co-transfection group) and quantified expression of HIF-1α and VEGF-A protein normalized to β-actin (HIF-1α expression in SOCS3 transfection group vs. non-transfection group, P<0.05; HIF-1α expression in SOCS3 transfection group vs. SOCS3+HIF-1α co-transfection group, P<0.05; VEGF-A expression in SOCS3 transfection group vs. non-transfection group, P<0.05; VEGF-A expression in SOCS3 transfection group vs. SOCS3+HIF-1α co-transfection group, P<0.05) (B) Growth curves of cells in SOCS3 transfection and SOCS3+HIF-1α co-transfection groups. Co-transfection with SOCS3 and HIF-1α significantly promoted the growth rate of the cells (NCI-H446/SOCS3 group vs. NCI-H446/SOCS3+HIF-1α group, P<0.05). (C) CD34 expression in the three groups (magnification, ×200). Immunofluorescence staining showed that following transfection with SOCS3, CD34 expression intensity was significantly decreased, while it was significantly intensified following upregulation of SOCS3 and HIF-1α. SOCS3, suppressor of cytokine signaling 3; VEGF, vascular endothelial growth factor; SCLC, small cell lung cancer; HIF-1α, hypoxia-inducible factor-1α.

Previous studies by our group demonstrated that HIF-1α enhances the proliferative and angiogenic potential of SCLC cells by regulating functional genes, including VEGF-A and interleukin-6 (1,2). From a previous microarray analysis, it was identified that the two other members of the SOCS family, SOCS1 and SOCS2, were also regulated by HIF-1α (1). However, the mutual regulation between SOCS3 and HIF-1α has not been previously reported. In the present study, it was found that following transfection with Ad5-SOCS3, SOCS3 expression was upregulated, but HIF-1α expression was downregulated; cell proliferation as well as VEGF-A and CD34 expression were also inhibited (Fig. 3A–C). Following co-transfection with SOCS3 and HIF-1α, HIF-1α expression, cell proliferation, VEGF-A and CD34 expression were all significantly upregulated compared with those following transfection with Ad5-SOCS3 only (Fig. 3A–C).

### SOCS3 inhibits HIF-1α expression through the Akt pathway, but not the STAT3 pathway

**Figure 4:**
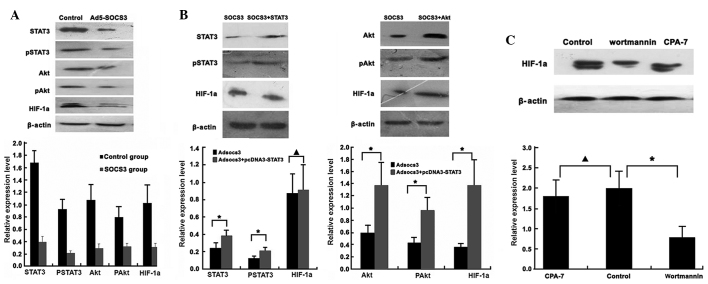
SOCS3 inhibits HIF-1α expression through the Akt pathway but not the STAT3 pathway. Protein levels of HIF-1α, STAT3, Akt and the phosphorylated, activated forms pSTAT3 and pAkt were assessed using western blot analysis. Representative images of three independent experiments and quantified levels normalized to β-actin are shown. (A) Following transfection of NCI-H446 cells with Ad5-SOCS3, the expression levels of HIF-1α, STAT3, Akt as well as pSTAT3 and pAkt decreased significantly (P<0.05, Ad5-SOCS3 group vs, control group). (B) In NCI-H446 co-transfected with Ad5-SOCS3 and pcDNA3-STAT3, STAT3 and pSTAT3 expression were upregulated (^*^P<0.05), while HIF-1α expression levels exhibited no significant change (^▲^P>0.05). However, when co-transfected with pcDNA3-Akt not only Akt and pAkt, but also HIF-1α expression levels were upregulated (^*^P<0.05). (C) In NCI-H446 cells treated with the STAT3 inhibitor CPA-7, HIF-1α expression levels exhibited no significant change (^▲^P>0.05). By contrast, when treated with Akt inhibitor wortmannin, HIF-1α expression levels were markedly inhibited (^*^P<0.05). SOCS3, suppressor of cytokine signaling 3; HIF-1α, hypoxia-inducible factor-1α; STAT3, signal transducer and transcription activator 3; p, phosphorylated.

As it is well-established that SOCS3 negatively regulates the JAK/STAT signaling pathway, SOCS3 was suggested to have a role in the Akt-mediated signaling pathway. To examine the underlying molecular mechanism by which SOCS3 inhibited HIF-1α expression in SCLC cells, the effect of SOCS3 on Akt and STAT3 phosphorylation as well as HIF-1α expression was assessed in NCI-H446 cells using western blot analysis. When SOCS3 was exogenously expressed in NCI-H446 cells, HIF-1α expression was inhibited and tyrosine phosphorylation of Akt and STAT3 as well as the protein levels of Akt and STAT3 were decreased (Fig. 4A). In addition, pcDNA3-STAT3 or pcDNA3-Akt was transfected into cells modified by Ad-SOCS3. It was found that along with the increased expression of Akt, rather than STAT3, HIF-1α expression was upregulated (Fig. 4B). Treatment of cells with wortmannin inhibited the Akt protein, including pAkt, and the expression of HIF-1α was also inhibited. However, treatment with CPA-7 decreased levels of STAT3 protein, including pSTAT3, but the expression of HIF-1α was not markedly inhibited (Fig. 4C). This suggested that SOCS3 inhibited HIF-1α expression in HCI-H446 cells through the Akt pathway but not the STAT3 pathway.

### Targeting Akt may inhibit the proliferative and angiogenic potential of SCLC cells

**Figure 5:**
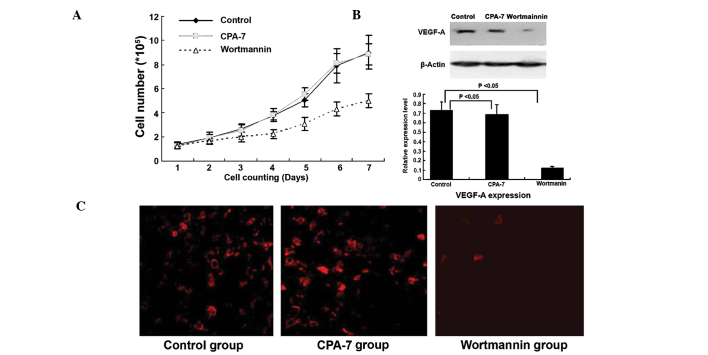
Targeting Akt inhibits the proliferation and angiogenic potential of SCLC cells. (A) Growth curves of cells in the control, CPA-7 and wortmannin groups. Following treatment with the Akt inhibitor wortmannin, the growth rate was significantly decreased; however, when treated with STAT3 inhibitor CPA-7, the growth curve was vimilar to that of the control group (NCI-H446/wortmannin group vs. NCI-H446/control group, P<0.05; NCI-H446/CPA-7 group vs. NCI-H446/control group, P>0.05). (B) Western blot analysis of VEGF-A protein expression. Representative images of three independent experiments and quantified results normalized to β-actin are shown. Following treatment with wortmannin, VEGF-A expression was significantly inhibited, but following treatment with CPA-7, VEGF-A expression exhibited no significant change (NCI-H446/wortmannin group vs. NCI-H446/control group, P<0.05; NCI-H446/CPA-7 group vs. NCI-H446/control group, P>0.05). (C) CD34 expression in three groups (magnification, x200). Immunofluorescence staining showed that following treatment with wortmannin, the CD34 expression was significantly decreased, while following treatment with CPA-7, CD34 expression was not significantly change. VEGF, vascular endothelial growth factor; SCLC, small cell lung cancer.

A previous study confirmed that JAK/STAT3 and PI3K/Akt pathways were involved in inhibiting the proliferative and angiogenic potential of tumor cells (21). The present study found that targeting Akt but not STAT3 inhibited SCLC cell growth and angiogenesis. Wortmannin-treatment of NCI-H446 cells significantly inhibited the growth rate and also CD34 and VEGF expression were inhibited. However, following the treatment with CPA-7, it was found that growth rate, CD34 and VEGF expression were not significantly inhibited (Fig. 5A–C).

### Upregulation of SOCS3 inhibits tumor growth in vivo

**Figure 6:**
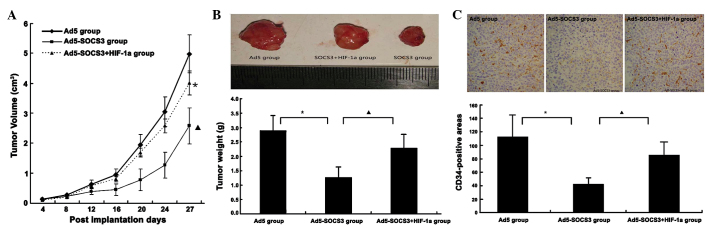
SOCS3 causes growth suppression and angiogenesis of NCI-H446 cells *in vivo* and HIF-1α is required in this process. (A) NCI-H446 cells were transfected with empty Ad5 vector, Ad5-SOCS3 or Ad5-SOCS3+HIF-1α. Subsequently, transfected cells (1×10^7^) of the three groups were subcutaneously injected into mice to form transplantation tumors. The growth curve shows that compared with that of the Ad5 group, the growth rate of Ad5-SOCS3 cells was decreased, particularly from 21 days after inoculation (Ad5-SOCS3 group vs. Ad5 group, ^*^P<0.05; n=11). Compared with that of the Ad5-SOCS3 group, the growth rate in the Ad5-SOCS3+HIF-1α co-transfection group was increased, particularly from 18 days after inoculation (Ad5-SOCS3+HIF-1α group vs. Ad5-SOCS3 group, ^▲^P<0.05, n=11). (B) Following sacrification of the mice 30 days after inoculation, tumor weight was significantly decreased in the Ad5-SOCS3 group (Ad5-SOCS3 group vs. Ad5 group, ^*^P<0.05, n=11) as compared with that in the Ad5 group, but following co-transfection with HIF-1α, the tumor weight increased (Ad5-SOCS3+HIF-1α group vs. Ad5-SOCS3 group, ^▲^P<0.05, n=11). (C) Representative microscopic images of CD34 antibody-stained tumor sections (magnification, ×10). The bar graph shows the mean neovascular densities ± standard deviation. Images of the entire area of each tumor were captured and analyzed. Results of immunohistochemical semiquantitative analysis showed that the number of CD34-positive areas was decreased following transfection with SOCS3 as compared with that in the empty vector-transfected group (^*^P<0.05), while this effect was significantly reduced following co-transfection with HIF-1α (Ad5-SOCS3+HIF-1α group vs. Ad5-SOCS3 group, ^▲^P<0.05). Ad5, tumor-specific replication-defective adenovirus type 5; SOCS3, suppressor of cytokine signaling, 3; HIF-1α, hypoxia-inducible factor-1α.

**Figure 7:**
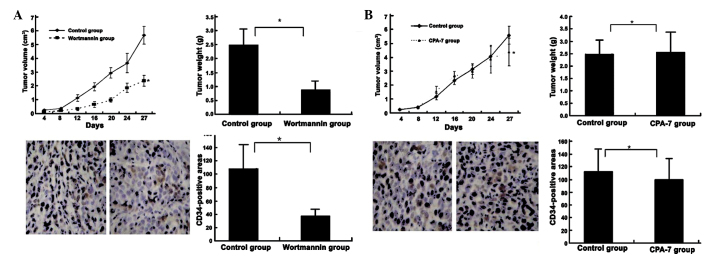
Blocking Akt but not STAT3 inhibits tumor growth and angiogenesis. (A) Targeting Akt in human tumors inhibited tumor growth and was accompanied by a reduction in tumor angiogenesis (CD34-positive areas). Nude mice bearing subcutaneous NCI-H446 tumors were treated with Akt inhibitor wortmannin at 3 mg/kg twice weekly. Compared with that in the control group, tumor growth in the wortmannin group was significantly decreased. Following sacrification of mice 30 days after inoculation, tumor weight was significantly decreased in the wortmannin group compared with that in the control group. Immunohistochemical analysis showed that CD34-positive areas were also significantly decreased in the wortmannin group as compared with those in the control group (wortmannin group vs. control group, ^*^P<0.05). (B) Inhibition of STAT3 in human tumors exhibited no significant inhibitory effect on tumor growth and angiogenesis. Nude mice bearing subcutaneous NCI-H446 tumors were treated with STAT3 inhibitor CPA-7, at 5 mg/kg twice weekly, which did not significantly affect tumor volume, weight and CD34-positive areas (CPA-7 group vs. control group, ^#^P>0.05). Values are expressed as the mean ± standard deviation. STAT3, signal transducer and activator of transcription 3.

As it was demonstrated that transfection with Ad5-SOCS3 produced a stably expressing cell line, these cells were subcutaneously injected into nude mice to form tumors. Compared with the tumor formed by cells transfected with the empty vector Ad5, tumors overexpressing SOCS3 exhibited a lower growth rate and lower rate of angiogenesis (Fig. 6A–C). These results demonstrated that SOCS3 is a tumor suppressor and inhibits the proliferative and angiogenic potential of SCLC cells. In a further *in vivo* experiment, the tumor formed by cells co-transfected with SOCS3 and HIF-1α exhibited a higher growth rate and level of angiogenesis compared with that of cells transfected with Ad5-SOCS3 only (Fig. 6A–C). This demonstrated that the inhibition of the proliferative and angiogenic potential of SCLC cells may be realized through the inhibition of HIF-1α expression. In another experiment, wortmannin and CPA-7 were injected for intervention of the subcutaneous transplantation tumor. In accordance with the results of the *in vitro* experiment, following wortmannin injection, the growth rate and angio-genesis of tumors were significantly inhibited, whilst CPA-7 injection had no significant effect (Fig. 7A and B). These results demonstrated that Akt is the key signaling pathway involved not only in HIF-1α expression, but also the proliferative and angiogenic potential of SCLC cells.

## Discussion

The tumor microenvironment is characterized by hypoxia and nutrient deprivation, which leads to genetic and epigenetic adaptation of cell clones, therefore enabling its invasive and metastatic nature. The adaptation of tumor cells to hypoxia makes them more difficult to treat and highly resistant to therapy (22). An important part of this process is the adaptation of gene products as a response to hypoxia and evidence suggested that a number of these hypoxia-regulated genes are mediated by HIF-1α (23). HIF-1α is involved in the response to hypoxia through oxygen homeostasis and also in myocardial, brain and retinal ischemia, pulmonary hypertension, preeclampsia, intrauterine growth, retardation and cancer (24). It has a crucial role in physiological homeostatic and etiopathological mechanisms. HIF-1α acts as a target factor for cancer therapeutics, as its function is regulated by growth factors and genetic abnormalities involved in tumor progression (25). It was identified that HIF-1α levels were adapted in SCLC cells to maintain a high rate of proliferation; however, the increased cell proliferation may induce an increased expression of HIF-1α (26). HIF-1α may be an effective therapeutic target for SCLC and inhibiting the expression of HIF-1α may be a suitable approach for developing molecular targeted therapies.

A previous study has confirmed that SOCS3 may significantly inhibit the proliferation of lung cancer cells *in vitro* and indicated that SOCS3 may act as an anti-oncogene involved in the development of tumors (10). In addition, SOCS3 may regulate the movement and migration of tumor cells (27). Hypermethylation-mediated silencing of SOCS3 has been identified in multiple types of cancer (20). Restoration of SOCS3 expression may be realized by demethylation or ectogenic SOCS3 gene transfer. He *et al* (28) reported that promoter hypermethylation-mediated silencing was found in human non-small cell lung cancer samples and several other tumor cell lines. Restoring SOCS3 expression through demethylation in such cancer cells may successfully suppress tumorigenesis. Shouda *et al* (29) reported that exogenous SOCS3 may suppress malignant fibrous histiocytoma tumor progression. In the present study, the methylation levels of SOCS3 were high in SCLC cells and SOCS3 expression was only moderately detected in NCI-H446 cells. When treated with the demethylation agent 5-aza-2′-deoxycytidine, SOCS3 mRNA levels were significantly upregulated but no marked changed in protein levels were observed. Therefore, it was hypothesized that the material cause was protein degradation and the avoidance of SOCS3 protein degradation during the experimental process would be a key priority in future investigation. However, transfection with ectogenic genes through adenovirus vectors enabled stable SCOS3 expression at the protein level, and therefore, through *in vivo* and *in vitro* study, it was found that SOCS3 inhibited the proliferation and angiogenesis of SCLC cells and these biological effects were achieved through the regulation of HIF-1α expression.

Previous studies have confirmed that expression of SOCS3 may be induced by a variety of cytokines and growth factors and directly antagonizes the STAT3-mediated signaling pathway as a classic negative feedback loop (30,31). A number of studies have demonstrated that other intracellular signaling cascades, including Ras/Erk1/2 (13) and PI3K/Akt (32), are also regulated by SOCS3, and their marked and persistent activation has been implicated in tumorigenesis. For the signaling pathway, the Jak/STAT and PI3K/Akt are two parallel pathways mediating functions of numerous receptor and non-receptor tyrosine kinases, including EGFR, Her-2 and c-Src (33). Yamasaki *et al* (34) found that Jak/STAT3 and PI3K/Akt were the main signaling pathways to regulate the expression of HIF-1α in numerous types of cancer. Xu *et al* (19) found that targeting STAT3 with STAT3 small interfering (si)RNA inhibited MCF-7 breast cancer cell proliferation induced by Jak/STAT and PI3K/Akt pathways, as transfection with STAT3 siRNA blocked not only STAT3 expression but also Akt expression. Yang *et al* (35) also hypothesized that curcumin blocked the proliferation of SCLC cells through the Jak/STAT signalling pathway to achieve its therapeutic effects. By contrast, the results of the present study differed from those of previous studies; it was found that although SOCS3 inhibited STAT3 expression, this regulation had no effect on the HIF-1α levels in SCLC cells, which was achieved through inhibition of Akt by SOCS3. In addition, the results of the present study demonstrated that targeting Akt, not STAT3, may inhibit the proliferative and angiogenic potential of SCLC cells. Therefore, it was hypothesized that SOCS3 as a therapeutic factor may inhibit the proliferation and HIF-1α expression in SCLC cells, which proceeded via the PI3K/Akt signaling pathway instead of the Jak/STAT signaling pathway, which has been the focus of investigation in previous years.

SCLC represents a malignant and particularly aggressive form of cancer with marked proliferation and a poor prognosis. Previous studies have mainly focused on radiotherapy and chemotherapy, but recently, molecular-targeted therapy has aroused increasing interest. For this, an effective therapeutic factor and a significant therapeutic target are important. SOCS3 was initially introduced as the therapeutic factor for SCLC and it was confirmed that SOCS3 may downregulate HIF-1α expression to inhibit the proliferation of SCLC cells. To date, there is no data delineating the signaling pathway involved in the SOCS3 regulation of HIF-1α expression in SCLC cells; however, it is hypothesized that the PI3K/AKT pathway may be involved. Therefore, the present study examined the regulation of biological activity by SOCS3, including migration, invasion, angiogenesis and the involved molecular mechanism.

In conclusion, the present study showed that SOCS3 inhibited the proliferative and angiogenic potential of NCI-H446 cells and HIF-1α was necessary in this process. A negative role of SOCS3 in Akt signaling, rather than STAT3 signaling to block HIF-1α expression as well as a previously unidentified regulatory mechanism for Akt function were observed. These results provided a theoretical basis for targeting SOCS3 for the treatment of SCLC.

## Figures and Tables

**Table I:** Methylation-polymerase chain reaction conditions and primer sequences.

Primer	Sequences	Tm (°C)	Length (bp)
Methylation primers	Sense: 5′-TTCGAGGTGTTCGAGTAGTC-3′ Antisense: 5′-AACGATCTTC CGACA AAAAT-3′	65	310
Unmethylation primers	Sense: 5′-TTTTTTGAGGTGTTTGAGTAGTT-3′ Antisense: 5′-AACAAT CTTCCAACAAAAATACT-3′		

Tm, annealing temperature; Length, the number of base pairs in the PCR products.

**Table II:** Polymerase chain reaction conditions and primer sequences

Gene	Primer	Tm (°C)	Length (bp)
SOCS3	Sense: 5′-GCTGGCGAAGGAAATGGT-3′ Antisense: 5′-GGAGCCTAGGGTGAAAGATG-3′	62	346
β-actin	Sense: 5′-CCAAGGCCAACCGCGAGAAGATGAC-3′ Antisense: 5′-AGGGTACATGGTGGTGGCGCCAGAC-3′	65	587

Tm, annealing temperature; Length, the number of bp in the PCR products.
